# Amyloid-β interrupts canonical Sonic hedgehog signaling by distorting primary cilia structure

**DOI:** 10.1186/s13630-018-0059-y

**Published:** 2018-08-17

**Authors:** Anna G. Vorobyeva, Aleister J. Saunders

**Affiliations:** Department of Biology, Drexel University, Philadelphia, PA USA

**Keywords:** Alzheimer’s disease (AD), Amyloid precursor protein (APP), Amyloid-β (Aβ), Sonic hedgehog (Shh), Primary cilia

## Abstract

**Background:**

Primary cilia are small non-motile microtubule and cell membrane protrusions expressed on most vertebrate cells, including cortical and hippocampal neurons. These small organelles serve as sensory structures sampling the extracellular environment and reprogramming the transcriptional machinery in response to environmental change. Primary cilia are decorated with a variety of receptor proteins and are necessary for specific signaling cascades such as the Sonic hedgehog (Shh) pathway. Disrupting cilia structure or function results in a spectrum of diseases collectively referred to as ciliopathies. Common to human ciliopathies is cognitive impairment, a symptom also observed in Alzheimer’s disease (AD). One hallmark of AD is accumulation of senile plaques composed of neurotoxic Amyloid-β (Aβ) peptide. The Aβ peptide is generated by the proteolytic cleavage of the amyloid precursor protein (APP). We set out to determine if Aβ affects primary cilia structure and the Shh signaling cascade.

**Methods:**

We utilized in vitro cell-based assays in combination with fluorescent confocal microscopy to address our study goals. Shh signaling and cilia structure was studied using two different cell lines, mouse NIH3T3 and human HeLa cells. To investigate how Aβ levels affect Shh signaling and cilia structure in these cells, we utilized naturally secreted Aβ as well as synthetic Aβ. Effects on Shh signaling were assessed by luciferase activity while cilia structure was analyzed by fluorescent microscopy.

**Results:**

Here, we report that APP localizes to primary cilia and Aβ treatment results in distorted primary cilia structure. In addition, we demonstrate that Aβ treatment interrupts canonical Shh signal transduction.

**Conclusions:**

Overall, our study illustrates that Aβ can alter primary cilia structure suggesting that elevated Aβ levels, like those observed in AD patients, could have similar effects on neuronal primary cilia in the brain. Additionally, our study suggests that Aβ impairs the Shh signaling pathway. Together our findings shed light on two novel targets for future AD therapeutics.

**Electronic supplementary material:**

The online version of this article (10.1186/s13630-018-0059-y) contains supplementary material, which is available to authorized users.

## Background

Elevated levels of Amyloid-β (Aβ) peptide initiates a cascade of events that ultimately result in Alzheimer’s disease (AD) [1–4]. Aβ is a small (4 kDa) neurotoxic peptide that can oligomerize into higher order structures. In AD, these oligomers interrupt neuronal activity resulting in synaptic dysfunction and eventually lead to cognitive decline [5–10]. Aβ is produced by sequential β- and γ-secretase proteolytic cleavage of the amyloid precursor protein (APP) [11–17]. APP function is not well understood, however, studies that shed light on the physiological role of Aβ in non-diseased brains show that Aβ plays a role in synaptic transmission and is involved in learning and memory formation [18–22]. For this reason, it is critical to maintain adequate soluble Aβ levels that support synaptic plasticity while not exceeding the pro-amyloidosis threshold. In our recent report, we discovered that cyclopamine, a Sonic hedgehog (Shh) signaling inhibitor, decreases Aβ generation by modulating γ-secretase-mediated cleavage of APP [17].

The Sonic hedgehog (Shh) signaling pathway is best known for its pivotal role in development and neurogenesis [23–28]. More recent reports identified active Shh signaling in the adult brain [27–29]. Specifically, components of the Shh signaling pathway are expressed in the adult hippocampal dentate gyrus, a brain region profoundly affected by Alzheimer’s disease.

The canonical Shh signaling cascade relies on the primary cilium for signal transduction [25, 29–34]. Virtually all mammalian cells bear a primary cilium [35, 36]. This conservation across cell types suggests that cilia play critical role(s) in cellular function. In humans, when cilia structure is disrupted, significant cellular and organ dysfunction is observed. All ciliopathies share one phenotype, cognitive impairment [37–42]. These findings suggest that neuronal cilia may house signaling cascades critical for learning and memory [43, 44] and therefore, may be important in AD pathogenesis.

At the primary cilium, Shh signaling is initiated by binding of the secreted Shh peptide to the patched 1 (Ptch1) receptor, resulting in disinhibition of smoothened (Smo), a G protein-coupled receptor (GPCR). Active Smo triggers a signaling cascade that ultimately results in Gli-mediated transcriptional regulation of downstream genes [45–49]. In mammals, this canonical Shh signaling pathway requires primary cilia [25, 29–34]. Impaired Shh signaling results in similar phenotypes as those observed in patients with disrupted cilia structure or function [37–42]. The aforementioned discoveries and our previous finding that the Shh signaling inhibitor, cyclopamine, alters APP metabolism and Aβ levels, prompted us to investigate a possible molecular cross-talk between Shh signaling, Aβ, and primary cilia.

In the current study, we evaluated whether increased Aβ levels disrupt primary cilia structure and the mammalian Sonic hedgehog signaling pathway. Using immunofluorescence microscopy, we detected Aβ induced changes in cilia structure. Furthermore, we found that Aβ treatment inhibits Shh signaling in NIH3T3 cells. Finally, we detected that APP localizes to the primary cilium with the Hedgehog signaling component Smo. This suggests that APP may have a functional role in the cilium and that distorted cilia structure and impaired canonical Shh signaling could be contributing factors to AD neuropathology.

## Methods

### Antibodies, plasmids and reagents

Antibodies were obtained from the following: rabbit APP C-terminus A8717, mouse anti-acetylated tubulin (Sigma: 1:1000). Fluorescent secondary antibodies included the AlexaFluor 488, 594, 649 (Jackson Immunoresearch Laboratories: 1:250). Cyclopamine (5 μM) was purchased from LC Laboratories, L-685,458 (2 μM) and DMSO from Sigma, SAG (100 nM) from Calbiochem, and human Aβ_42_ peptide (0.1–5 μM) [50, 51] from Tocris R&D Systems. pcDNA3.1-EGFP-Smo and pcDNA3.1-Cherry-APP695 were used for overexpression studies.

### Cell culture

HeLa and NIH3T3 cells were maintained at 37 °C, 5% CO_2_ in complete DMEM (Corning) supplemented with 10% FBS (Atlanta Biologicals), 100 units/ml penicillin and 100 μg/ml streptomycin (Corning), 2 mM l-glutamine (Corning). Cells were grown to 80% confluence and serum starved (0.5% FBS DMEM) for 24 h to induce ciliogenesis and subsequently pharmacologically treated or genetically manipulated. For pharmacological treatment, drugs were diluted in 0.5% FBS DMEM. For genetic overexpression experiments, cells were transfected using TurboFect Transfection Reagent (Thermo Scientific) according to the manufacturer’s protocol. For Aβ containing conditioned media, stable HEK293 APP_695_^swd^ or naïve HEK293 cells were grown in 150 mm dish to 100% confluence and culture media was replaced with 15 ml 0.5% FBS DMEM to cover the cells. Cells were cultured with or without supplemental L-685,458 for an additional 48 h then the media was collected and cleared by a brief 1000×*g* centrifugation. For NIH3T3 treatment with biological Aβ, conditioned media was diluted 1:2 in fresh 0.5% FBS DMEM prior to addition to confluent NIH3T3s. Stable HEK293 APP_695_^swd^ cells were generated by overexpressing the Cherry-APP_695_^swd^ mammalian expression construct in HEK293 cells and selecting single cell colonies by supplementing the growth media with G418 (Geneticin; 400 μg/ml) for 14 days.

### LDH cytotoxicity assay

Levels of LDH were measured according to manufacturer’s protocol (Roche). Briefly, conditioned media were collected from treated cells, 1:1 ratio of this media to LDH master mix was incubated at ambient temperature for 30 min. Samples were analyzed using spectrophotometer.

### Luciferase assay

Stable NIH3T3 Shh-Light2 (kind gift from N. Dahmane University of Pennsylvania, PA, USA) cells were grown to 90% confluence in 96-well plate, then serum starved for 24 h and exposed to indicate pharmacological agents for an additional 24 h.

Lysates were collected using 1XGLB Lysis Buffer (Promega) and subject to Bright-Glo Luciferase assay (Promega) according to manufacturer’s protocol and samples were analyzed using Promega Luminescent plate reader. Firefly Luciferase luminescence values were normalized to cell number determined by SYBR (molecular probes) green assay.

### Immunofluorescence

Cells were fixed using 4% PFA, 0.1% Triton-X-100, blocked in 2% BSA for 30 min and incubated with primary antibodies over night at 4 °C. Cells were rinsed with PBS and stained with secondary antibodies at room temperature for 1 h, washed with PBS and mounted (Vectashield with DAPI, VectorLabs). Sequential Z-stack images were taken (63 × oil objective, 2–5 × zoom, 30–50 slices were imaged at 0.25 μm step size, 1024 × 1024 pixels) using Olympus Fluoview FV1000 inverted confocal microscope (Drexel University Cell Imaging Center). Quantification of 3D confocal image stacks was accomplished using Volocity Image analysis software (PerkinElmer). The following Volocity settings were most reliable and reproducible. Randomly selected fields (> 10 fields/cover slip) were used for quantification: an object protocol in Volocity was created to identify ROIs (cilia) in max projection stacked images by gating size (> 1 μm^3^ min, < 7 μm^3^ max), ≥ 0.2 μm diameter, and fluorescence intensity thresholds (500 min–4095 max) [52].

### Statistical analysis

All graphs and diagrams represent mean values ± standard error of all triplicates from at least three independent experiments. ANOVA and two-tailed Student’s *t* tests were used when appropriate to compare three or two treatment groups, respectively, and calculate significance from at least three independent experiments (*p < *0.05*, **p < *0.01*, ***p < *0.005*).

## Results

### Aβ decreases primary cilia length and frequency in NIH3T3 cells

**Fig. 1:**
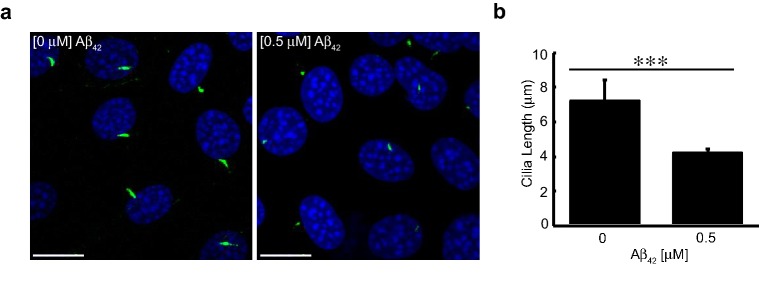
Synthetic Amyloid-β decreases cilia length. **a** Confocal immunofluorescence analysis of naïve NIH3T3 cells treated with synthetic Aβ_42_ peptide for 24 h. Cells were stained with acetylated tubulin and > 50 cilia per condition analyzed. Scale bar represents 20 μm. **b** Quantification of naïve NIH3T3 cells exposed to synthetic human Aβ_42_ for 24 h. Cilia length was analyzed using an anti-acetylated tubulin antibody and immunofluorescence, 30–50 cells were analyzed using Volocity 3D analysis software. Values denote mean ± standard errors of the means. Student’s *t* test was used for statistical analysis: ***p < *0.005*, **p < *0.01*, *p < *0.05*

**Fig. 2:**
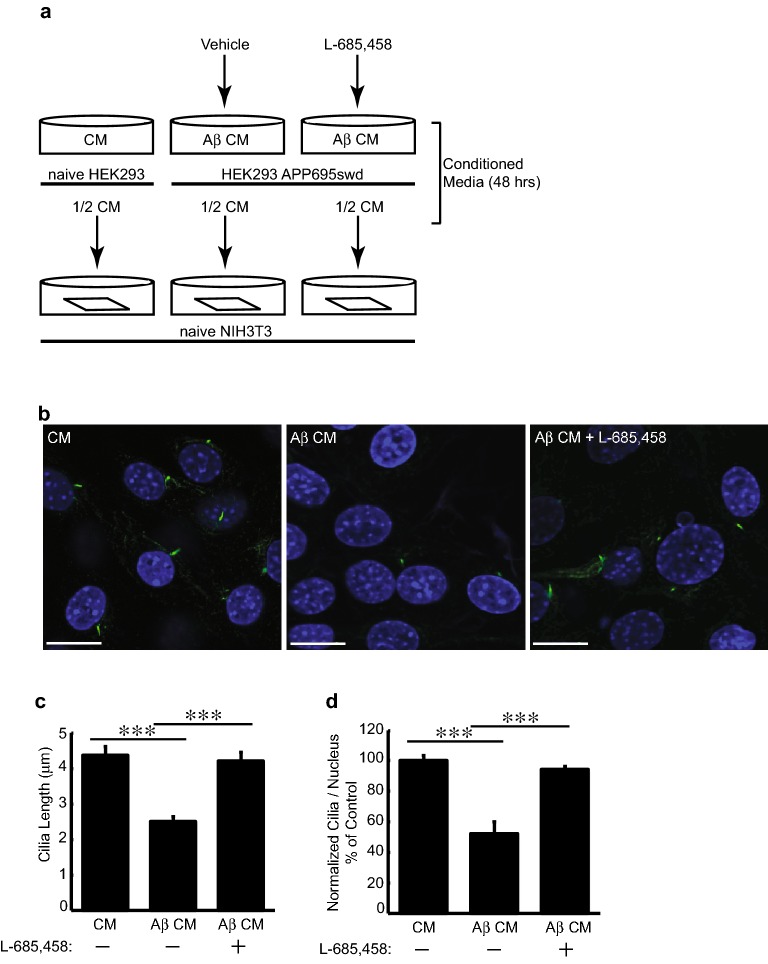
Secreted Amyloid-β treatment decreases cilia length. **a** Representation of experimental approach to generate secreted neurotoxic Aβ. Naïve and stable HEK293_APP_^swd^ cells were cultured and conditioned media collected for NIH3T3 treatment. Conditioned media (CM) collected from stable HEK293_APP_^swd^ treated with vehicle control (DMSO) is designated as “Aβ CM” while stable cells treated with 2 μM L-685,458 is designated as “Aβ CM + L685,458”. Conditioned media was diluted 1:1 with fresh DMEM immediately prior to NIH3T3 treatment. **b** Confocal 3D analysis of naïve NIH3T3 cells treated with vehicle control CM, Aβ CM, or Aβ CM_L685,458 for 24 h. Cells were stained with acetylated tubulin and > 50 cilia per condition analyzed. Scale bar represents 20 μm. **c**, **d** Bar graph representing quantification of cilia length and frequency. Values denote mean ± standard errors of the means. Student’s *t* test was used for statistical analysis: ***p < *0.005*, **p < *0.01*, *p < *0.05*

To determine whether elevated Aβ levels affect primary cilia structure, we exposed cells to increasing concentrations of extracellular Aβ and examined cilia structure by immunofluorescence microscopy using an anti-acetylated tubulin antibody, an established primary cilia marker. Mouse NIH3T3 fibroblasts were serum-starved for 24 h, to induce cilium formation, then exposed to various Aβ concentrations [50, 52]. Exposure of these cells to 0.5 μM synthetic human Aβ_42_ resulted in significant reduction in primary cilia length (43%) (Fig. 1a, b). As validation, 3D voxel analysis (using Volocity; see “Methods”) also provided data indicating significant reduction in cilia volume and surface area which were consistent with reduced cilia length in cells treated with Aβ (data not shown). To ensure that the effects were not limited to synthetic Aβ, we exposed cells to conditioned media containing cellularly secreted Aβ. To generate this biologically synthesized Aβ, we cultured HEK293 cells that stably overexpress APP_695_^swd^ and collected the conditioned culture media (Fig. 2a). APP_695_^swd^ cells harbor the APP Swedish mutation (K670N/M671L), which increases β-secretase cleavage, resulting in elevated levels of Aβ secretion [53]. We used conditioned media from naïve HEK cells as a negative control. Compared to cells that were exposed to conditioned media from naïve cells, cells treated with conditioned media from APP_695_^swd^ cells, showed a significant decrease in cilia length (47%) (Fig. 2b, c). Additionally, the percentage of ciliated cells decreased by 47.5% (*p *= 4.095 × 10^−6^) upon treatment with Aβ-conditioned media (Fig. 2d). Our results indicate that extracellular Aβ decreases the number of cilia per cell and alters cilia structure. These degenerative-like effects were rescued when cells were exposed to conditioned media collected from APP_695_^swd^ HEKs grown in the presence of 2 μM L-685,458 (Fig. 2a–d). L-685,458 is a commonly used γ-secretase transition-state inhibitor which inhibits Aβ generation. Taken together, these results indicate that extracellular Aβ has adverse effects on the maintenance and structure of primary cilia.

### Aβ disrupts canonical Sonic hedgehog signaling

**Fig. 3:**
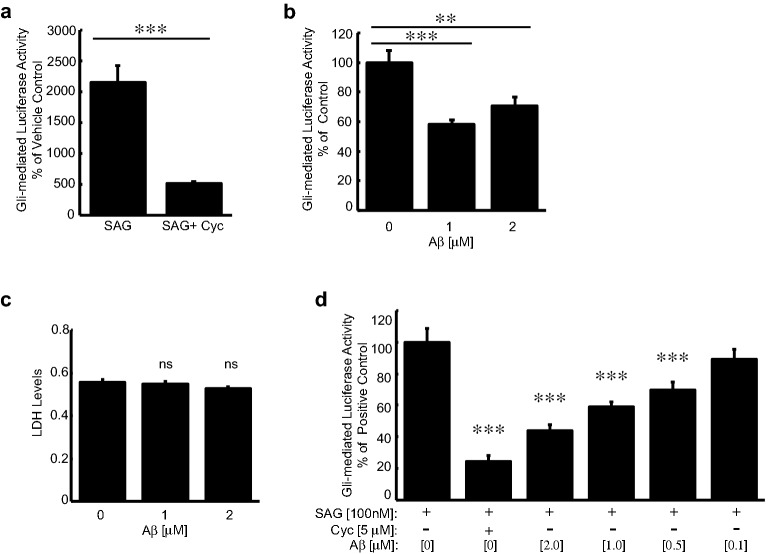
Neurotoxic Amyloid-β disrupts canonical Sonic hedgehog signal transduction. **a** Gli-mediated luciferase activity of NIH3T3 Shh-Light2 cells treated with 100 nM SAG or in combination with 5 μM cyclopamine for 24 h. Quantification represents Gli-mediated luciferase activity normalized to vehicle control treated cells. **b** Gli-mediated luciferase activity of NIH3T3 Shh-Light2 cells treated with indicated concentrations of synthetic human Aβ_42_ peptide for 24 h. **c** LDH cytotoxicity analysis of NIH3T3 Shh-Light2 cells treated with Aβ_42_ peptide for 24 h. **d** Gli-mediated luciferase activity of NIH3T3 Shh-Light2 cells treated with SAG and increasing concentrations of Aβ_42_ peptide for 24 h. Values denote mean ± standard errors of the means. Student’s *t* test was used for statistical analysis: ***p < *0.005*, **p < *0.01*, *p < *0.05*

Vertebrate canonical Shh signaling requires proper primary cilia structure. Since we observed that Aβ disrupts cilia structure, we tested whether Aβ would also alter canonical Shh signaling. We utilized NIH3T3 Shh-Light2 cells, a commonly used Shh reporter system. NIH3T3 Shh-Light2 cells stably express the Gli-responsive luciferase construct [54]. Upon Shh activation with the agonist SAG, endogenous Gli transcription factors enhance luciferase expression while the Shh antagonist cyclopamine, decreases Gli-mediated luciferase expression (Fig. 3a).

We observed that exposing these reporter cells to Aβ decreased the Gli-mediated luciferase activity regardless of whether Shh signaling was simulated by SAG or not. In the absence of SAG, we exposed Shh reporter cells to varying levels of synthetic Aβ_42_ and detected a robust decrease in basal Gli-mediated luciferase activity. Specifically, Gli-mediated luciferase activity decreased by 42% and 30% in cells treated with 1 μM and 2 μM Aβ_42_, respectively (Fig. 3b). One possibility is that the observed decrease in canonical Shh signaling could be due to Aβ-mediated cell death. To address this, we utilized the lactate dehydrogenase (LDH) cytotoxicity assay, which measures cellular cytotoxicity and cytolysis. Since we did not detect changes in LDH levels across all treatments, our results indicate that the observed Aβ-mediated effects on Gli-mediated luciferase activity are not due to cytotoxicity (Fig. 3c).

We observed a more substantial decrease in Gli-mediated luciferase activity when cells were simultaneously treated with Aβ and SAG. We observed a 56% decrease in cells treated with 2 μM Aβ_42_ and an 11% decrease in cells treated with as little as 100 nM synthetic Aβ_42_ (Fig. 3d). Consistent with canonical Shh signaling, luciferase expression decreased significantly in cells co-treated with SAG and cyclopamine as well as the combination of SAG and synthetic Aβ_42_. These results show that elevated Aβ interrupts steady-state and agonist-induced canonical Shh signaling. These results correlate with defective cilia structure and maintenance, which was previously shown to alter Shh signaling [26, 29, 34].

### APP localizes to primary cilia

**Fig. 4:**
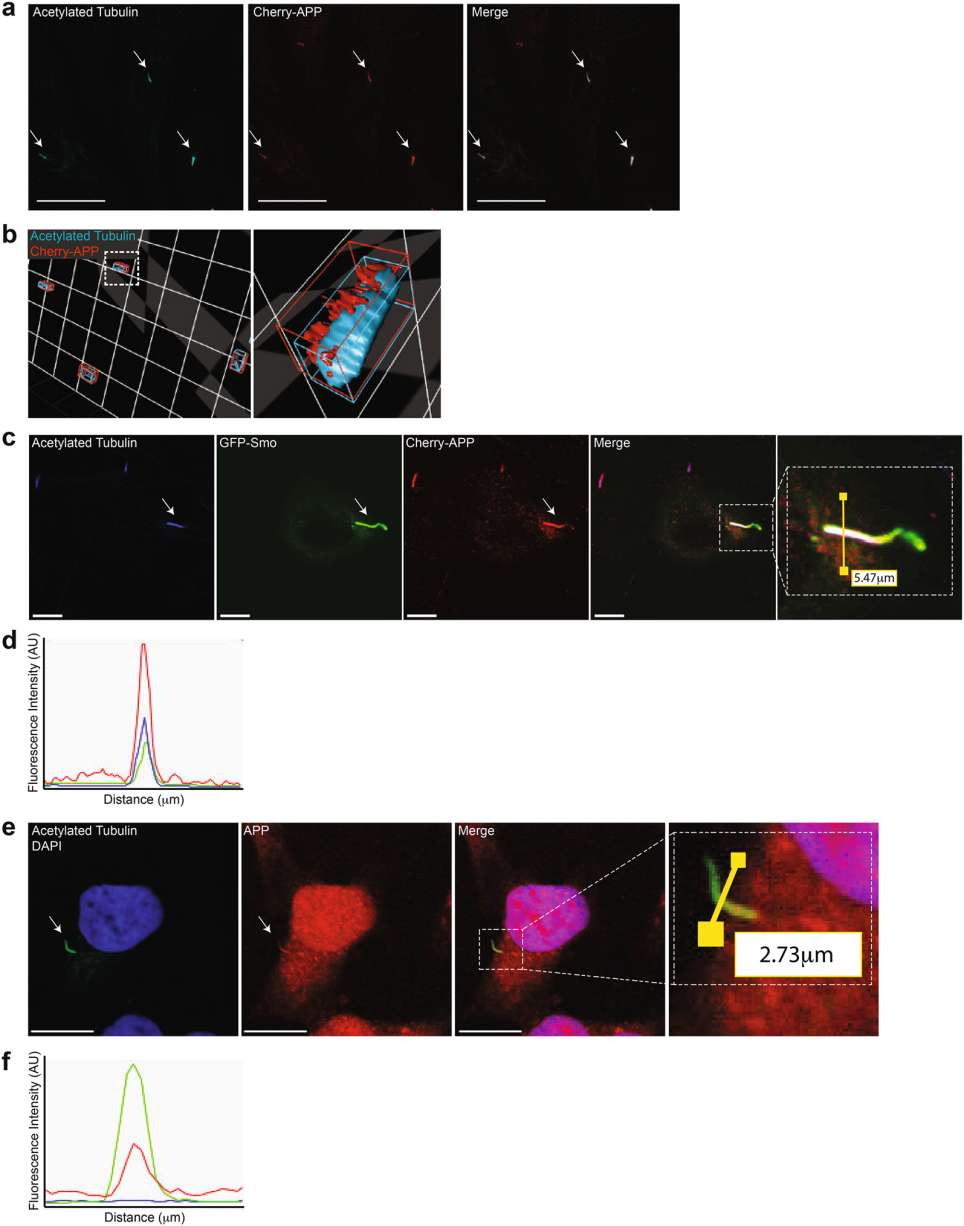
Amyloid precursor protein localizes to primary cilia of NIH3T3 and HeLa cells. **a** Confocal analysis of overexpressed Cherry-APP localization in NIH3T3 cells stained using anti-acetylated tubulin antibody. Scale bar, 25 μm. **b** Compartmentalization analysis of acetylated tubulin (blue), Cherry-APP (red) using Volocity 3D imaging analysis software of the previous image in **a**. **c**, **d** Localization analysis of fluorescence intensity of NIH3T3 cells overexpressing Cherry-APP and GFP-Smo constructs. **d** Fluorescence intensities of acetylated tubulin (blue), GFP-Smo (green), and Cherry-APP (red) though the 5.47 μm cross section depicted in right-most panel of **c**. **e** Confocal immunofluorescence analysis of naïve HeLa cells co-stained with anti-acetylated tubulin and C-terminal APP antibody. Scale bar, 7 μm. (**f**) Fluorescence intensities of nuclei (blue), acetylated tubulin (green), and endogenous APP (red) though the 2.73 μm cross section depicted in right-most panel of **e**

Since canonical Shh signaling requires primary cilia, we used immunofluorescence to investigate if the amyloid precursor protein (APP) localizes to the primary cilium. We utilized two established markers of primary cilia, acetylated tubulin and Smo, to identify cilia [55–57]. A commercially available antibody is available for acetylated tubulin, however, a reliable Smo antibody is currently unavailable, and thus it is difficult to analyze endogenous Smo. To overcome this shortcoming, researchers routinely transiently overexpress human GFP-Smo to identify cilia [55–57]. Here, we transiently co-overexpressed human Cherry-APP and GFP-Smo in NIH3T3 cells. These cells were also stained with an antibody to acetylated tubulin. These experiments revealed that APP localizes to the primary cilium with acetylated tubulin (Fig. 4a–d) and GFP-Smo (Fig. 4c, d). To further validate this finding and rule out possible artifacts due to APP overexpression, we investigated whether endogenous APP localized to primary cilia in HeLa cells. Using the A8717 APP-specific antibody we again identified endogenous APP-positive primary cilia (Pearson’s correlation coefficient *r *= 0.91) (Fig. 4e, f, Additional file 1). Interestingly, Additional file 1 reveals APP accumulation at the ciliary basal compartment and a less intense APP signal at ciliary distal tip was detected. The latter is consistent with one proposed localization mechanism for proteins destined to primary cilia. Briefly, golgi-derived cilia-bound vesicles are trafficked to cilia basal body complex. One function of the cilia basal body complex is to sort and facilitate cilia-specific transmembrane cargo for further cilia intraflagellar transport (IFT) (reviewed in [58, 59]). Therefore, one could speculate that a portion of APP packaged at the Golgi arrives at ciliary basal body complex to gain access to primary cilia membrane for further sensory functions. A recent report by Kohli and colleagues used a combination of biotinylation and mass spec to characterize proteins localized to the primary mouse IMCD3 cell cilia [60]. The authors list APP in their full report but do not validate APP with secondary methods. Therefore, to our knowledge, we are first to validate that APP protein localizes to primary cilia and can be observed within this organelle together with the Hedgehog signaling component Smo. Since APP function is largely elusive, the aforementioned results could suggest that APP may have a functional role in the primary cilium.

## Discussion

Sonic hedgehog (Shh) signaling requires an intact cilia structure, since Shh signaling fails in cells with disrupted cilia structure [30–34]. We provide two lines of evidence suggesting Shh signaling and cilia are novel targets for Alzheimer’s disease (AD). First, we previously demonstrated an intriguing yet indirect connection between Shh signaling and APP metabolism. Specifically, the Shh antagonist, cyclopamine, reduced γ-secretase mediated APP cleavage and resulted in decreased Aβ levels [17]. We did not illustrate the direct mechanism by which Sonic hedgehog inhibition could alter APP metabolism, we are still investigating this question. Hedgehog signaling is complex, with canonical and non-canonical signaling mechanisms described [61, 62]. Knowing the critical role that primary cilia play in Hedgehog signaling, we wanted to investigate a possible link between Aβ and Hedgehog signaling at the level of the primary cilium.

Our second line of evidence is our current results demonstrating that increased Aβ levels disrupt cilia structure and inhibit canonical Shh signaling. The Shh signaling pathway is known for its role in development, neurogenesis and cell survival [26, 63, 64]. With our findings, one could postulate that increased Aβ levels observed in AD leads to cilia degeneration, which ultimately impairs Shh signaling and precludes Shh-mediated neuronal survival. Consistent with this possibility is the observation that impaired ciliogenesis leads to decreased synaptogenesis and neuronal maturation [65, 66].

**Fig. 5:**
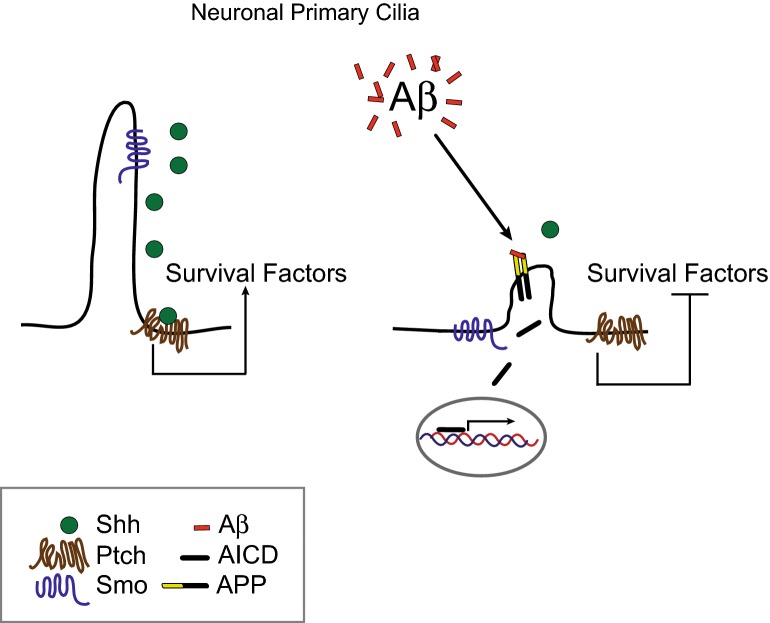
Cartoon illustration of a possible mechanism for APP sensing Aβ at the primary cilium. On the right, disrupted primary cilium structure due to increased Aβ levels leads to interrupted Sonic hedgehog signaling, abolished secretion of survival factors, and increased AICD-mediated transcriptional activation pro-amyloidogenic genes and the Shh negative regulator *PTCH1*

Diseases associated with abnormal cilia structure are collectively referred to as ciliopathies and affect a wide range of organ systems resulting in a spectrum of phenotypes and symptoms [35–42, 67]. Interestingly, cognitive impairment is a common symptom among ciliopathies [67, 68]. Human neuronal tissue and cultured cells express primary cilia [69–76]. Recent studies utilizing genetically modified mouse models where primary cilia are conditionally disrupted have demonstrated that neuronal primary cilia are critical for neurodevelopment and maintenance of neurogenesis in the adult hippocampal and cortical structures [26, 34, 65, 77]. These findings illustrate that disrupting cilia structure subsequently leads to alteration of important signaling pathways that are required for mechanisms such as neurogenesis and overall cellular homeostasis. Our results indicate that (i) exposure to elevated Aβ levels distorts cilia structure, (ii) Aβ can disrupt Shh signaling, and (iii) that APP localizes to primary cilia. This last finding begs the question of what functional role could APP have at the primary cilium. Interestingly, Fogel and colleagues demonstrated it is possible that APP could dimerize at the plasma membrane and bind Aβ peptide [78]. This study suggests APP may function as a receptor for its own peptide, Aβ. Therefore, we tested the hypothesis that overexpressing wild-type APP, which unlike the Swedish mutated APP results in normal Aβ levels, would enhance the cells’ sensitivity to additional Aβ treatment. Consistent with our hypothesis, we observed that APP overexpressing cells began to appear damaged and unhealthy within 1–3 h post treatment with various Aβ concentrations. This degenerative-like cellular phenotype was not observed in cells overexpressing empty-vector control (unpublished results) and only detected 24 h post treatment suggesting this sensitivity is specific to APP overexpression. Since cilia are sensory organelles decorated with an assortment of receptor proteins such as G protein-coupled and growth factor receptors, one can speculate that APP could also function as a receptor sensing extracellular Aβ levels at the primary cilium. This could lead to downstream signaling via the APP intracellular domain (AICD) (Fig. 5). Previous reports suggest AICD modulates Shh signaling by regulating transcriptional activation of *PTCH1*, a negative regulator of Shh signaling, further indicating a relationship between Shh signaling and APP [79, 80]. Our observation that Aβ affects cilia structure and cilia-dependent Shh signaling further supports the notion that APP localizes to cilia for a specific function which could be to act as an Aβ-sensing receptor to mediate downstream cell survival signals.

Recent reports suggest cilia and type 3 adenylyl cyclase are also involved in cognitive functions such as learning and memory [43, 44]. Progressive decline in memory is observed in Alzheimer’s disease which led us to hypothesize that cilia may be affected in AD brains. In support of our hypothesis, Chakravarthy and colleagues recently reported diminished cilia length of hippocampal dentate granule cells in an AD mouse model [81]. Consistent with the latter, our results suggest that increased Aβ levels could also be disruptive to neuronal cilia and potentially a mechanism underlying neuronal loss and cognitive decline observed in AD. It will be interesting to examine histological cilia structure in AD and non-AD human postmortem brain tissue at various stages of the disease. Interestingly, He et al. provided insight into the canonical Shh signaling pathway using APP23 transgenic mouse model and compared the results to biochemical analysis of healthy controls and Alzheimer’s patient human brain tissue results [82]. The authors consistently observed elevated Shh peptide protein levels and Smo levels in APP23 mice of a variety of ages. Levels of other canonical Shh signaling cascade components, such as Ptch1, Ptch2, and the Gli proteins varied significantly with mouse age. Smo, however, was consistently elevated in all ages of APP23 mice. In addition, consistent with our cell culture data, the authors observed decreased Ptch–Gli1 mediated signaling in cultured mouse glial precursor cells and decreased neurogenesis in aged APP23 mice.

## Conclusions

In this study, we discovered that Amyloid-β, which plays a central role in Alzheimer’s disease pathogenesis, (i) distorts primary cilia structure, (ii) disrupts canonical Sonic hedgehog (Shh) signaling and (iii) that its precursor, APP, localizes to the primary cilium along with smoothened (Smo) a known cilia resident Shh signaling component. We also rescued the cilia degenerative-like morphological changes by pharmacologically suppressing Amyloid-β production. The latter result confirmed that the degenerative-like effects on cilia structure and Shh signaling are specifically due to elevated Amyloid-β levels. Moreover, to our knowledge, this is the first report to validate that APP protein localizes to the primary cilium by immunofluorescence microscopy. Together, our findings suggest new AD risk factors and therapeutic targets, as well as a novel putative function for APP and its role in ciliopathy-associated cognitive impairment.

## Additional file

**Additional file 1.** 3D rendering of naive HeLa cells fixed and costained for endogenous APP (red) using the APP C-terminal A8717 antibody and acetylated tubulin (green) antibody to detect cilia. DAPI (blue) was used to detect cell nuclei. Video rendering was made using Volocity imaging analysis software.

## Abbreviations

AD: Alzheimer’s disease
APP: amyloid precursor protein
Aβ: Amyloid-β
GPCR: G-protein-coupled receptor
Ptch1: patched receptor 1
Shh: Sonic hedgehog
Smo: smoothened

## References

[1] Hardy J, Allsop D (1991). Amyloid deposits as the central event in the etiology of Alzheimer’s disease. Trends Pharmacol Sci.

[2] Selkoe DJ (1991). The molecular pathology of Alzheimer’s disease. Neuron.

[3] Hardy J, Selkoe DJ (2002). The amyloid hypothesis of Alzheimer’s disease: progress and problems on the road to therapeutics. Science.

[4] Bertram L, Tanzi RE (2005). The genetic epidemiology of neurodegenerative disease. J Clin Invest..

[5] Hsia AY, Masliah E, McConlogue L, Yu GQ, Tatsuno G, Hu K, Kholodenko D, Malenka RC, Nicoll RA, Mucke L (1999). Plaque-independent disruption of neural circuits in Alzheimer’s disease mouse models. Proc Natl Acad Sci USA.

[6] Stern EA, Bacskai BJ, Hickey GA, Attenello FJ, Lombardo JA, Hyman BT (2004). Cortical synaptic integration in vivo is disrupted by amyloid-beta plaques. J Neurosci.

[7] Berezov TT, Kudinova NV, Kudonov AP (2005). The role of Alzheimer’s amyloid plaques in the mechanisms of neuronal synaptic plasticity disturbance. Vestn Ross Akad Med Nauk.

[8] Kelly BL, Ferreira A (2007). Beta-amyloid disrupted synaptic vesicle endocytosis in cultured hippocampal neurons. Neuroscience.

[9] Spires-Jones TL, Meyer-Luehmann M, Osetek JD, Jones PB, Stern EA, Bacskai BJ, Hyman BT (2007). Impaired spine stability underlies plaque-related spine loss in an Alzheimer’s disease mouse model. Am J Pathol.

[10] Potter PE, Rauschkolb PK, Pandya Y, Sue LI, Sabbagh MN, Walker DG, Beach TG (2011). Pre- and post-synaptic cortical cholinergic deficits are proportional to amyloid plaque presence and density at preclinical stages of Alzheimer’s disease. Acta Neuropathol.

[11] Scheuner D, Eckman C, Jensen M, Song X, Citron M, Suzuki N, Bird TD, Hardy J, Hutton M, Kukull W, Larson E, Levy-Lahad E, Viitanen M, Peskind E, Poorkaj P, Schellenberg G, Tanzi R, Wasco W, Lennfelt L, Selkoe D, Younkin S (1996). Secreted amyloid beta-protein similar to that in the senile plaques of Alzheimer’s disease is increased in vivo by the presenilin 1 and 2 and APP mutations linked to familial Alzheimer’s disease. Nat Med.

[12] Kim TW, Tanzi RE (1997). Presenilins and Alzheimer’s disease. Curr Opin Neurobiol.

[13] De Strooper B, Saftig P, Craessaerts K, Vanderstichele H, Guhde G, Annaert WM, Von Figura K, Van Leuven F (1998). Deficiency of presenilin-1 inhibits the normal cleavage of amyloid precursor protein. Nature.

[14] Haass C, De Strooper B (1999). The presenilins in Alzheimer’s disease—proteolysis holds the key. Science.

[15] Vassar R, Bennett BD, Babu-Khan S, Kahn S, Mendiaz EA, Denis P, Teplow DB, Ross S, Amarante P, Leoloff R, Luo Y, Fisher S, Fuller J, Edenson S, Lile J, Jarosinski MA, Biere AL, Curran E, Burgess T, Louis JC, Collins F, Treanor J, Rogers G, Citron M (1999). Beta-secretase cleavage of Alzheimer’s amyloid precursor protein by the transmembrane aspartic protease BACE. Science.

[16] Wolfe MS, Xia W, Moore CL, Leatherwood DD, Ostaszewski B, Rahmati T, Donkor IO, Selkoe DJ (1999). Peptidomimetic probes and molecular modeling suggest that Alzheimer’s gamma-secretase is an intramembrane-cleaving aspartyl protease. Biochemistry.

[17] Vorobyeva AG, Lee R, Miller S, Longen C, Sharoni M, Kandelwal PJ, Kim FJ, Marenda DR, Saunders AJ (2014). Cyclopamine modulates γ-secretase-mediated cleavage of amyloid precursor protein by altering its subcellular trafficking and lysosomal degradation. J Biol Chem.

[18] Seubert P, Vigo-Pelfrey C, Esch F, Lee M, Dovey H, Davis D, Sinha S, Schlossmacher M, Whaley J, Swindlehurst E (1992). Isolation and quantification of soluble Alzheimer’s beta-peptide from biological fluids. Nature.

[19] Pearson HA, Peers C (2006). Physiological roles for amyloid beta peptides. J Physiol.

[20] Kumar DK, Choi SH, Washicosky KJ, Eimer WA, Tucker S, Ghofrani J, Lefkowitz A, McColl G, Goldstein LE, Tanzi RE, Moir RD (2016). Amyloid-β peptide protects against microbial infection in mouse and worm models of Alzheimer’s disease. Sci Transl Med..

[21] Saura CA, Choi SY, Beglopoulos V, Malkani S, Zhang D, Shankaranarayana Rao BS, Chattarji S, Kelleher RJ, Kandel ER, Duff K, Kirkwood A, Shen J (2004). Loss of presenilin function causes impairments of memory and synaptic plasticity followed by age-dependent neurodegeneration. Neuron.

[22] Puzzo D, Privitera L, Leznik E, Fà M, Staniszewki A, Palmeri A, Arancio O (2008). Picomolar amyloid-beta positively modulates synaptic plasticity and memory in hippocampus. J Neurosci.

[23] Lee JJ, von Kessler DP, Parks S, Beachy PA (1992). Secretion and localized transcription suggest a role in positional signaling for products of the segmentation gene hedgehog. Cell.

[24] Echelard Y, Epstein DJ, St-Jacques B, Shen L, Mohler J, McMahon JA, McMahon AP (1993). Sonic hedgehog, a member of a family of putative signaling molecules, is implicated in the regulation of CNS polarity. Cell.

[25] Varjosalo M, Taipale J (2008). Hedgehog: functions and mechanisms. Genes Dev.

[26] Breunig JJ, Sarkisian MR, Arellano JI, Morozov YM, Ayoub AE, Sojitra S, Wang B, Flavell RA, Rakic P, Town T (2008). Primary cilia regulate hippocampal neurogenesis by mediating Sonic hedgehog signaling. Proc Natl Acad Sci USA.

[27] Ihrie PA, Shah JK, Harwell CC, Levine JH, Guinto CD, Lezamera M, Kriegstein AP, Alvarez-Buylla A (2011). Persistent Sonic hedgehog signaling in adult brain determines neural stem cell positional identity. Neuron.

[28] Alvarez-Buylla A, Ihrie RA (2014). Sonic hedgehog signaling in the postnatal brain. Semin Cell Dev Biol.

[29] Han YG, Spassky N, Romaguera-Ros M, Garcia-Verdugo JM, Aguilar A, Schneider-Maunoury S, Alvarez-Buylla A (2008). Hedgehog signaling and primary cilia are required for the formation of adult neural stem cells. Nat Neurosci.

[30] Houde C, Dickinson RJ, Houtzager VM, Cullum R, Montpetit R, Metzler M, Simpson EM, Roy S, Hayden MR, Hoodless PA, Nicholson DW (2006). Hippi is essential for node cilia assembly and Sonic hedgehog signaling. Dev Biol.

[31] Huangfu D, Anderson KV (2005). Cilia and Hedgehog responsiveness in the mouse. Proc Natl Acad Sci USA.

[32] Huangfu D, Liu A, Rakeman AS, Murcia NS, Niswander L, Anderson KV (2003). Hedgehog signaling in the mouse requires intraflagellar transport proteins. Nature.

[33] Corbit KC, Aanstad P, Singla V, Norman AR, Stainier DY, Reiter JF (2005). Vertebrate smoothened functions at the primary cilia. Nature.

[34] Town T, Breunig JJ, Sarkisian MP, Spilianakis C, Ayoub AE, Liu X, Ferrandino AF, Gallagher AR, Li MO, Rakic P, Flavell RA (2008). The stumpy gene is required for mammalian ciliogenesis. Proc Natl Acad Sci USA.

[35] Badano JL, Karsanis N (2006). Life without centrioles: cilia in the spotlight. Cell.

[36] Berbari NF, O’Connor AK, Haycraft CJ, Yoder BK (2009). The primary cilium as a complex signaling center. Curr Biol.

[37] Badano JL, Mitsuma N, Beales PL, Katsanis N (2006). The ciliopathies: an emerging class of human genetic disorders. Annu Rev Genomics Hum Genet.

[38] Sattar S, Gleeson JG (2011). The ciliopathies in neuronal development: a clinical approach to investigation of Joubert syndrome and Joubert syndrome-related disorders. Dev Med Child Neurol.

[39] Hildebrandt F, Benzing T, Katsanis N (2011). Cilipathies. N Engl J Med.

[40] Duldulao NA, Li J, Sun Z (2010). Cilia in cell signaling and human disorders. Protein Cell.

[41] Lee JE, Gleeson JG (2011). Cilia in the nervous system: linking cilia function and neurodevelopmental disorders. Curr Opin Neurol.

[42] Waters AM, Beales PL (2011). Ciliopathies: an expanding disease spectrum. Pediatr Nephrol.

[43] Berbari NF, Malarkey EB, Yazdi SM, McNair AD, Kippe JM, Croyle MJ, Kraft TW, Yoder BK (2014). Hippocampal and cortical primary cilia are required for aversive memory in mice. PLoS ONE.

[44] Wang Z, Phan T, Storm DR (2011). The type 3 adenylyl cyclase is required for novel object learning and extinction of contextual memory; role of cAMP signaling in primary cilia. J Neurosci.

[45] Sasaki H, Nishizaki Y, Hui C, Nakafuku M, Kondoh H (1999). Regulation of Gli2 and Gli3 activities by an amino-terminal repression domain: implication of Gli2 and Gli3 as primary mediators of Shh signaling. Development.

[46] Bai CB, Auerbach W, Lee JS, Stephen D, Joyner AL (2002). Gli2, but not Gli1, is required for initial Shh signaling and ectopic activation of the Shh pathway. Development.

[47] Taipale J, Cooper MK, Maiti T, Beachy PA (2002). Patched acts catalytically to suppress the activity of smoothened. Nature.

[48] Rohatgi R, Milenkovic L, Scott MP (2007). Patched1 regulates hedgehog signaling at the primary cilium. Science.

[49] Milenkovic L, Scott MP, Rohatgi R (2009). Lateral transport of smoothened from the plasma membrane to the membrane of the cilium. J Cell Biol.

[50] Lazarevic V, Fieńko S, Andres-Alonso M, Anni D, Ivanova D, Montenegro-Venegas C, Gundelfinger E, Cousin M, Fejtova A (2017). Physiological concentrations of amyloid beta regulate recycling of synaptic vesicles via Alpha7 acetylcholine receptor and CDK5/calcineurin signaling. Front Mol Neurosci.

[51] Kirouac L, Rajic AJ, Cribbs DH, Padmanabhan J (2017). Activation of Ras-ERK signaling and GSK-3 by amyloid precursor protein and amyloid beta facilitates neurodegeneration in Alzheimer’s disease. eNeuro..

[52] Farnum CE, Williams RM, Donnelly E (2009). Analyzing primary cilia by multiphoton microscopy. Methods Cell Biol.

[53] Citron M, Vigo-Pelfrey C, Teplow DB, Miller C, Schenk D, Johnston J, Winblad N, Venizelos N, Lannfelt L, Selkoe DJ (1994). Excessive production of amyloid beta-protein by peripheral cells of symptomatic and presymptomatic patients carrying the Swedish familial Alzheimer’s disease mutation. Proc Natl Acad Sci USA.

[54] Taipale J, Chen JK, Cooper MK, Wang B, Mann RK, Milenkovic L, Scott MP, Beachy PA (2000). Effects of oncogenic mutations in smoothened and patched can be reversed by cyclopamine. Nature.

[55] Bhattacharyya S, Rainey MA, Arya P, Mohapatra BC, Mushtaq I, Dutta S, George M, Storck MD, McComb RD, Muirhead D, Todd GL, Gould K, Datta K, Gelineau-van Waes J, Band V, Band H (2016). Endocytic recycling protein EHD1 regulates primary cilia morphogenesis and SHH signaling during neural tube development. Sci Rep.

[56] Joo K, Kim CG, Lee MS, Lee SH, Kim MJ, Kweon HS, Park WY, Kim CH, Gleeson JG, Kim J (2013). CCDC41 is required for ciliary vesicle docking to the mother centriole. Proc Natl Acad Sci USA.

[57] Wang Y, Zhou Z, Walsh CT, McMahon AP (2009). Selective translocation of intracellular smoothened to the primary cilium in response to Hedgehog pathway modulation. PNAS.

[58] Emmer BT, Maric D, Engman DM (2010). Molecular mechanisms of protein and lipid targeting to ciliary membranes. J Cell Sci.

[59] Jin H, Nachury MV (2009). The BBsome. Curr Biol.

[60] Kohli P, Höhne M, Jüngst C, Bertsch S, Ebert LK, Schauss AC, Benzing T, Rinschen MN, Schermer B (2017). The Ciliary membrane-associated proteome reveals actin-binding proteins as key components of cilia. EMBO Rep.

[61] Jenkins Dagan (2009). Hedgehog signaling: emerging evidence for non-canonical pathways. Cell Signal.

[62] Carballo GB, Honorato JR, de Lopes GPF (2018). Spohr TCLSE. A highlight on Sonic hedgehog pathway. Cell Commun Signal..

[63] Machold R, Hayashi S, Rutlin M, Muzumdar MD, Nery S, Corbin JG, Gritli-Linde A, Dellovade T, Porter JA, Rubin LL, Dudek H, McMahon AP, Fishell G (2003). Sonic hedgehog is required for progenitor cell maintenance in telencephalic stem cell niches. Neuron.

[64] Palma V, Lim DA, Dahmane N, Sànchez P, Brionne TC, Herzberg CD, Gitton Y, Carleton A, Alvarez-Buylla A, Ruiz i Altaba A (2005). Sonic hedgehog controls stem cell behavior in the postnatal and adult brain. Development..

[65] Kumamoto N, Gu Y, Wang J, Janoschka S, Takemaru K, Levine J, Ge S (2012). A role for primary cilia in glutamatergic synaptic integration of adult-born neurons. Nat Neurosci.

[66] Tissir F, Goffinet AM (2012). Cilia: conductors’ batons of neuronal maturation. Nat Neursci..

[67] Gitten J, Dede D, Fennell E, Quisling R, Maria BL (1998). Neurobehavioral development in Joubert syndrome. J Child Neurol.

[68] Guadiana SM, Parker AK, Filho GF, Sequeira A, Semple-Rowland S, Shaw G, Mandel RJ, Foster TC, Kumar A, Sarkisian MR (2016). Type 3 adenylyl cyclase and somatostatin receptor 3 expression persists in aged rat neocortical and hippocampal neuronal cilia. Front Aging Neurosci..

[69] Del Cerro MP, Snider RS (1969). The Purkinje cell cilium. Anat Rec.

[70] Mandl L, Megele R (1989). Primary cilia in normal human neocortical neurons. Z Mikrosk Anar Forsch..

[71] Händel M, Schulz S, Stanarius A, Schreff M, Erdrmann-Vourliotis M, Schmidt H, Wolf G, Hölt V (1999). Selective targeting of somatostatin receptor 3 to neuronal cilia. Neuroscience.

[72] Whitfield JF (2003). Primary cilium- is it an osteocyte’s strain-sensing flowmeter?. J Cell Biochem.

[73] Fuchs JL, Schwark HD (2004). Neuronal Primary cilia: a review. Cell Biol Int.

[74] Bishop GA, Berbari NF, Lewis J, Mykytyn K (2007). Type III adenylyl cyclase localizes to primary cilia throughout the adult mouse brain. J Comput Neurol..

[75] Berbari NF, Bishop GA, Askwith CC, Lewis JS, Mykytyn K (2007). Hippocampal neurons possess primary cilia in culture. J Neurosci Res.

[76] Arellano JI, Guadiana SM, Breunig JJ, Rakic P, Sarkisian MR (2012). Development and distribution of neuronal cilia in mouse neocortex. J Comp Neurol..

[77] Willaredt MA, Tasouri E, Tucker KL (2013). Primary cilia and forebrain development. Mech Dev.

[78] Fogel H, Frere S, Segev O, Bharill S, Shapira I, Gazit N, O’Malley T, Slomowitz E, Berdichevsky Y, Walsh DM, Isacoff EY, Hirsch JA, Slutsky I (2014). APP homodimers transduce an Amyloid-β-mediated increase in release probability at excitatory synapses. Cell Rep..

[79] Raychaudhuri M, Mukhopadhyay D (2010). AICD overexpression in neuro 2A cells regulates expression of PTCH1 and TRPC5. Int. J Alzheimer’s Dis..

[80] Trazzi S, Mitrugno VM, Valli E, Fuchs C, Rizzi S, Guidi S, Perini G, Bartesaghi R, Ciani E (2011). APP-dependent up-regulation of Ptch1 underlies proliferation impairment of neuronal precursors in Down syndrome. Hum Mol Genet.

[81] Chakravarthy B, Gaudet C, Mènard M, Brown L, Atkinson T, Laferla FM, Armato U, Dal Prà I, Whitfield J (2012). Reduction of the immunostainable length of the hippocampal dentate granule cells’ primary cilia in 3xAD-transgenic mice producing human Aβ(1–42) and tau. Biochem Biophys Res Commun.

[82] He P, Staufenbiel M, Li R, Shen Y (2014). Deficiency of patched 1-induced Gli1 signaling transduction results in astrogenesis in Swedish mutated APP transgenic mice. Hum Mol Genet.

